# Enhanced Spring Steel’s Strength Using Strain Assisted Tempering

**DOI:** 10.3390/ma15207354

**Published:** 2022-10-20

**Authors:** Zbyšek Nový, Pavel Salvetr, Jakub Kotous, Petr Motyčka, Aleksandr Gokhman, Črtomir Donik, Ján Džugan

**Affiliations:** 1 COMTES FHT a.s., Prumyslova 995, 334 41 Dobrany, Czech Republic; 2 South Ukrainian National Pedagogical University (SUNPU), Staroprotfrankivska 26, 65020 Odessa, Ukraine; 3 Institute of Metals and Technology (IMT), Lepi pot 11, 1000 Ljubljana, Slovenia

**Keywords:** medium carbon steel, tempering, strain, microstructure, mechanical properties, dilatometry

## Abstract

Spring steels are typical materials where enhancement of mechanical properties can save considerable mass for transport vehicles, in this way the consumption of fuel or electric energy can be decreased. A drastic change in both the resulting microstructure and mechanical properties could be achieved due to the inclusion of strain into the tempering process after quenching. The strain assisted tempering (SAT) technology was applied, i.e., the process of quenching and following a sequence of tempering operations alternating with strain operations. After the first tempering, controlled deformation by rotary swaging was carried out with a strain of 17% (strain rate is about 120 s^−1^). Considerably higher strength parameters after SAT compared to conventional quenching and tempering (QT) technology were nevertheless accompanied by enhanced notch toughness at the same time by the decrease of elongation and reduction of area. However, by optimizing the process it is was also possible to achieve acceptable values for those parameters. Remarkable differences are visible in resulting microstructures of compared samples, which were revealed by metallographic analysis and X-ray diffraction measurement. While the standard microstructure of tempered martensite with transition carbides was observed after QT processing, carbideless islands with nanotwins occurred in martensitic laths after SAT processing.

## 1. Introduction

Excellent mechanical properties of spring steel are attained by heat treatment or thermomechanical treatment, which effectively utilizes the potential of the elements present in the alloying system of the steel. Transformation strengthening achieved during quenching, precipitation strengthening and grain size strengthening achieved during tempering result in high yield strength, sometimes even above 2000 MPa. These hardenings, as well as a complex combination of plastic properties, such as elongation, reduction of area, toughness parameters and mainly fatigue properties, are affected due to microstructure features, such as the size of prior austenitic grains (PAG), the purity of PAG boundaries, the morphology of martensitic formations, residual austenite (RA) parameters, tempering precipitation characteristics and so on.

The essential factor for very high strength parameters is transformation strengthening, where the austenite face-centered cubic (fcc) lattice transforms due to the shear mechanism to a body-centered tetragonal (bct) lattice. This bct lattice does not have slip planes such as cubic lattice, and carbon in supersaturated interstitial solution prevents the slip. As a result, the stress field around solute atoms interacts with the stress field of dislocation, and the ability of the dislocations to move is significantly decreased [1].

The martensite formations of middle carbon low alloyed (MCLA) spring steel after quenching have a typical lath morphology. Prior austenite grains are divided into several packets with parallel blocks. These blocks are composed of laths arranged parallel to each other. Researchers [2] have proved the dominant role of the lath width on grain boundary strengthening of MCLA steel. The relationship between lath width and strength follows the Hall–Petch formula, indicating that lath width is the effective grain size (EGS) of strength in MCLA steel. This pattern was observed in steels with carbon content above 0.4 wt% [2].

It was established [3] that the hardness of the as-shocked iron increases with a decrease in the inter-twins spacing. Therefore, the latter was also considered as EGS.

The transition carbides precipitate during tempering at lower temperatures; the temperature range of their existence usually lies between 150 and 350 °C, while more stable cementite-like carbides precipitate in the range of 350–600 °C. The morphology of transition carbides in medium carbon steels seems to have characteristic features [4,5]. These carbides create typical rod-like or elongated plate-like formations, which have well-defined crystallography orientation in relation to the matrix crystal lattice. The width of such formations is usually 5–20 nm, while the length is hundreds of nm. The elongated transition carbides in the martensitic plate create a system of evenly spaced parallel formations. Such a system can spread—especially in the advanced precipitation stage—into the whole martensitic lath. Sometimes even two systems of parallel formations are visible in one martensitic lath. The stoichiometry of transition carbides of middle carbon low-alloyed steels is usually Fe_2_C or Fe_4_C.

On the other hand, the morphology of cementite is entirely different and primarily more varied. Cementite particles are usually more spheroidized, and if those are elongated, then only to a lesser extent. Furthermore, the localities of cementite carbides seem to be more diverse. They often occupy boundaries, e.g., martensite lath borders or prior austenite boundaries. Nevertheless, they nucleate and grow in the martensitic plates’ interior. The cementite-like carbides start to nucleate around 400 °C, and their dissolution begins above 650 °C [6].

The next possible type of carbide, which could be present after quenching and tempering, seems to be carbides of microalloying elements. Usually, these carbides arise during the processing before quenching and are stable during all process chains, including high-temperature tempering. These carbides are usually of spheroidized shape and very fine, with a diameter of around 5 nm [7].

The highest yield strength value can usually be detected after tempering at 250–350 °C, within a temperature range when transition carbides are stable. Within this temperature range, enough stress due to the remaining carbon in the supersaturated solid solution still remains in the microstructure. At the same time, the precipitation strengthening caused by the fine dispersion of the transition carbides culminates. A higher tempering temperature brings a strength decrease and an increase in ductility and toughness. An open question remains—looking primarily for the maximal yield strength—whether the deformation hardening could effectively enhance the values currently attained without a terminal decline of ductility and toughness parameters. Our efforts are motivated by the pursuit to store maximum energy in the elastic deformation of spring steel. According to the study [8], this effect can be achieved if the hardening process-quenching is followed by double tempering while a controlled strain is applied between each tempering operation. Pre-straining of materials introduces plastic deformation, which increases their resistance to flow. As a result, the nucleation and the propagation of the fatigue crack are delayed, thus increasing the fatigue life [9]. Pre-straining the annealed material removes the locked dislocations inherent in carbon steel [10]. The strain-induced martensite transformation and the dislocation density change during pre-straining may influence the diffusion of interstitial atoms [11]. Effects of pre-straining of the direct quenched samples (without tempering) are manifested by the division of polygonal ferrite grains by the dislocation cells [12].

The presented work is explicitly devoted to enhancing spring steels’ strength properties and providing satisfactory plastic properties through strain-assisted tempering (SAT) application. The next goal is the comparison of the properties after conventional quenching and tempering (QT) treatment and experimental SAT treatment. Investigation of mechanical properties is complemented by a detailed study of microstructure, dilatometry analysis, X-ray analysis and SAT.

## 2. Materials and Methods

The medium carbon spring steel BX classified as 54SiCr6 (W.nr. 1.7201) steel was melted in a vacuum induction furnace and cast into a 45 kg ingot. The chemical composition is given in Table 1. A Q4Tasman optical emission spectrometer (Bruker Elemental GmbH, Kalkar, Germany) was used for determination.

The ingot was brought to 1050 °C, hot rolled into a sheet with a thickness of 14 mm and air cooled. Normalization annealing was carried out at 850 °C for 40 min. Cylindrical samples of 13 mm in diameter and 120 mm in length were manufactured. After normalizing, two different processing followed. The first was conventional quenching followed by cooling at a rate of 20 °C/s and QT, and the second was quenching and SAT.

### 2.1. Quenching and Tempering (QT) Process

The quenching temperature was 900 °C with a holding time of 20 min. Next, the samples were quenched in oil followed by tempering in an electrical furnace from 200 to 400 °C for 120 min. The QT heat treatment of samples and their designation is documented in Table 2.

### 2.2. Quenching and Strain Assisted Tempering Process

**Figure 1:**
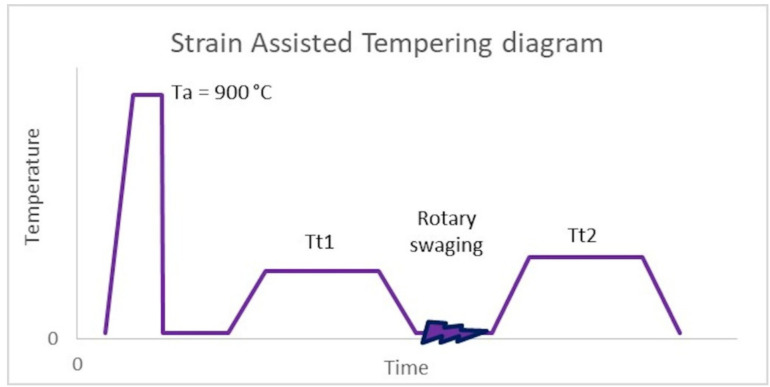
SAT diagram, Austenitization temperature (T_a_), 1st tempering temperature (T_t1_), 2nd tempering temperature (T_t2_).

SAT is a member of the group of improved thermo-mechanical processes. The quenching temperature was 900 °C with a holding time of 20 min. The samples were quenched in oil, followed by the first tempering in an electrical furnace at 200 or 250 °C. After the first tempering, controlled deformation by rotary swaging was performed with a strain of 17% (strain rate is about 120 s^−1^) (see Figure 1). The last step of SAT processing was the second tempering in an electric furnace, as shown in Table 3.

### 2.3. Methods

The metallography analysis, dilatometry, X-ray diffraction, tensile and Charpy impact tests were performed on all processed samples. 

**Figure 2:**
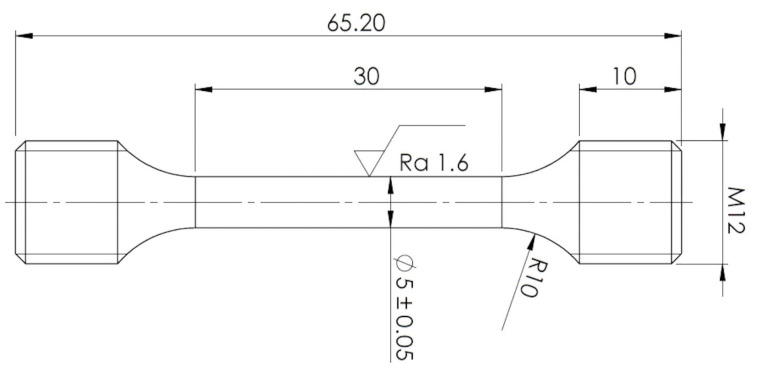
Specimen geometry for tensile test measurement.

Mechanical properties were examined at room temperature. Tensile tests were performed using a Zwick-Roell 250 servo-electric testing machine (ZwickRoell GmbH & Co. KG, Ulm, Germany). Elongations of specimens were measured using an Epsilon tensometric extensometer. Cylindrical tensile specimens were prepared from these various experimental conditions per ČSN EN ISO 6892-1 standard. Specimen geometry is shown in Figure 2. Four tensile specimens were performed for each experimental condition. In addition, Charpy impact tests were performed according to ISO 148-1 and EN ISO 14556 on an instrumented Charpy pendulum with an impact energy of 300 J.

The metallographic samples were prepared by mechanical grinding and polishing in the longitudinal direction of specimens. The microstructure was revealed by etching in 3% Nital, and observed using light microscopy (NIKON ECLIPSE MA200; NIKON, Tokyo, Japan), scanning electron microscopy-SEM (JEOL IT 500 HR; JEOL, Tokyo, Japan) and transmission electron microscopy (JEOL JEM-2200FS, JEOL 050422, JEOL, Tokyo, Japan).

X-ray diffraction (XRD) analysis was performed on electrolytically polished surfaces of samples on a Bruker Advance D8 diffractometer (Bruker AXS GmbH, Karlsruhe, Germany) with a cobalt anode (λ K_α1_ = 0.178897 nm). The increment was set to 0.02° in the range of 2Θ from 45° to 105° in Bragg–Brentano geometry. The dislocation densities were calculated from the martensite crystallographic planes of (110), (200) and (211) using the modified Williamson–Hall method [13].

Dilatometry was performed on an L75PT dilatometer (Linseis, Selb, Germany) with samples heated in a furnace for heating at lower heating rates. In addition, the L78 RITA quenching dilatometer (Linseis) was used for quenching and heating at 0.1 K/s and higher rates. The experiments were performed in pure nitrogen, and the specimens were held in quartz glass push rods with a contact force of 0.3 N.

## 3. Results

### 3.1. Results of Mechanical Testing

Yield strength (YS), ultimate strength (UTS), elongation (EL), area reduction (AR) and impact toughness (KCV) after conventional quenching and tempering up to 600 °C are given in Table 4.

The ultimate strength of QT samples up to a tempering temperature of 200 °C remains at similar levels of around 2400 MPa, and above this, it decreases with increasing tempering temperature. 

A significant maximum in the range of 250–350 °C of the yield strength was revealed, which is caused by the precipitation of fine transition carbides [1]. The plastic parameters show a continuously increasing trend from 250 to 400 °C. A rapid increase in AR, up to 40%, is observed. The important processing section lies in the tempering range from 200 to 400 °C. Here there is increasing steel plasticity, and high strength characteristics are maintained. This range was chosen for developing the processing based on the dynamic ageing of martensite. The results after SAT processing are presented in Table 5.

The first tempering temperature (T_t1_) of 200 °C was probably insufficient. In this case, the steel reached almost 3000 MPa in yield strength and ultimate strength after complete SAT processing, but the plasticity was practically exhausted, and the material showed minimum elongation. On the other hand, the T_t1_ of 250 °C leads to the preservation of better deformation characteristics. The results at T_t1_ 250 °C show excellent strength with relatively good elongation and notch toughness; the measured values are given in Table 5.

The values of about 4% EL, 20% AR and 20 J/cm^2^ KCV with a yield strength of about 2700–2850 MPa are impossible to achieve by conventional hardening.

Keeping the second tempering temperature (T_t2_) in the range of 250–350 °C, yield and tensile strength are close together and reach values above 2500 MPa. At the same time, the notch toughness maintains values close to 20 J/cm^2^ while samples after QT processing maintain lower KCV values in the 12–19 J/cm^2^. The increasing trend of the T_t2_ causes a reduction of both strength parameters; when the second tempering temperature reaches 400 °C, the strength parameters decrease to typical values around 2000 MPa. The influence of tempering time was tested on the SAT 250–300 samples, where the tempering time was prolonged in the range of 2–10 h. However, this brought about no substantial changes in strength nor plasticity or toughness.

**Figure 3:**
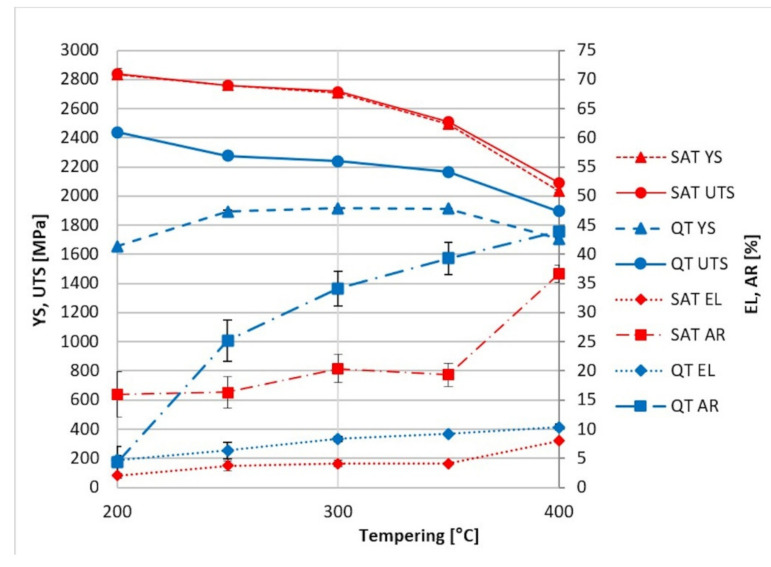
Comparison of tensile testing results from conventional QT (blue) and SAT processing (red).

Figure 3 compares the properties achieved by QT and SAT processing. It is evident that SAT, in which both temperings are kept below the cementite precipitation temperature, dramatically enhances the strength properties. The yield strength increases almost by half after QT processing. Furthermore, tensile strength is considerably higher after SAT, maximum values being above 2750 MPa. In this case, the percentage increase compared to QT is roughly 25%.

**Figure 4:**
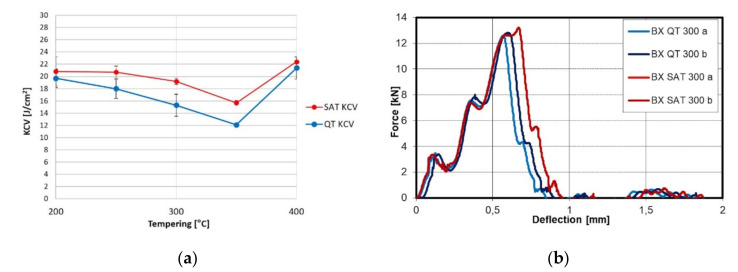
(**a**) KCV trend after conventional QT and SAT processing; (**b**) instrumental Charpy graph after conventional QT and SAT processing at 300 °C.

The relation of notch toughness to tempering temperature is shown in Figure 4. Increasing the temperature causes the growth of KCV; nevertheless, in the range of 200–400 °C, a drop is visible for the QT samples. This drop is almost eliminated after SAT processing. The force profile showed higher maximum force due to the strength characteristics after SAT. The bigger triangle plane means higher energy is required to destroy the sample.

### 3.2. Microstructural Analysis

**Figure 5:**
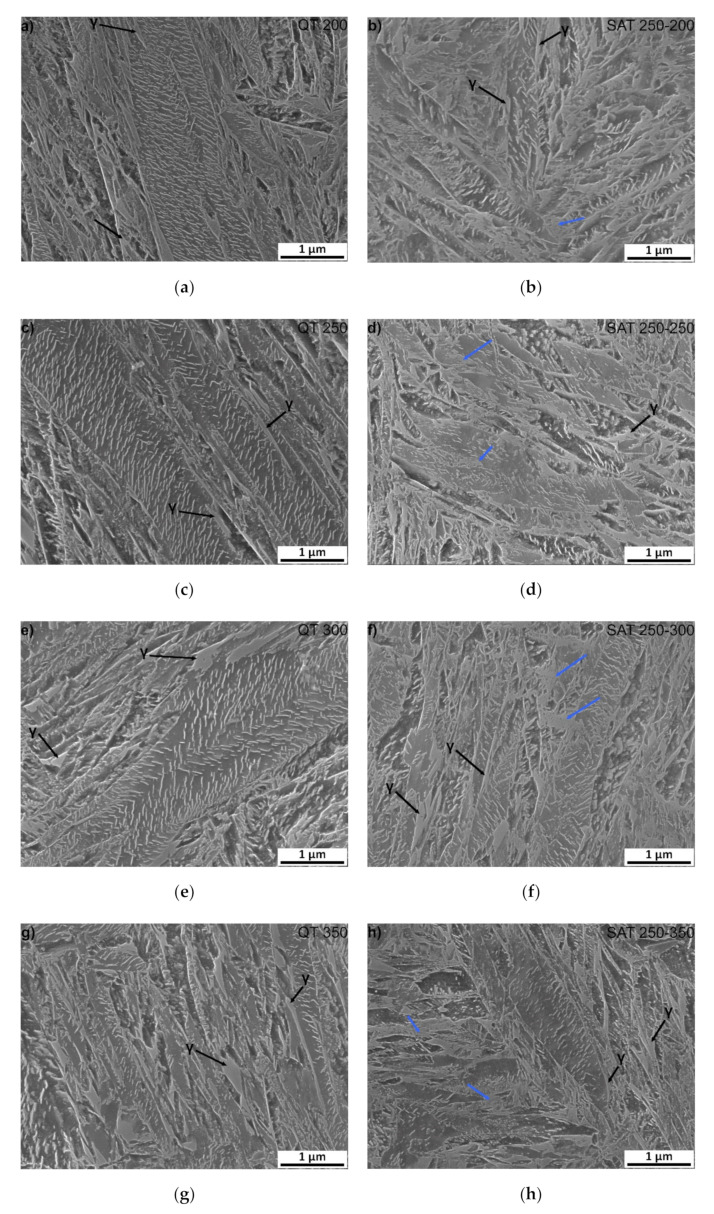
SEM microstructure of: (**a**) QT 200; (**b**) SAT 250–200; (**c**) QT 250; (**d**) SAT 250–250; (**e**) QT 300; (**f**) SAT 250–300; (**g**) QT 350 and (**h**) SAT 250–350. Blue arrows indicate bright islands with the lower etchability.

**Figure 6:**
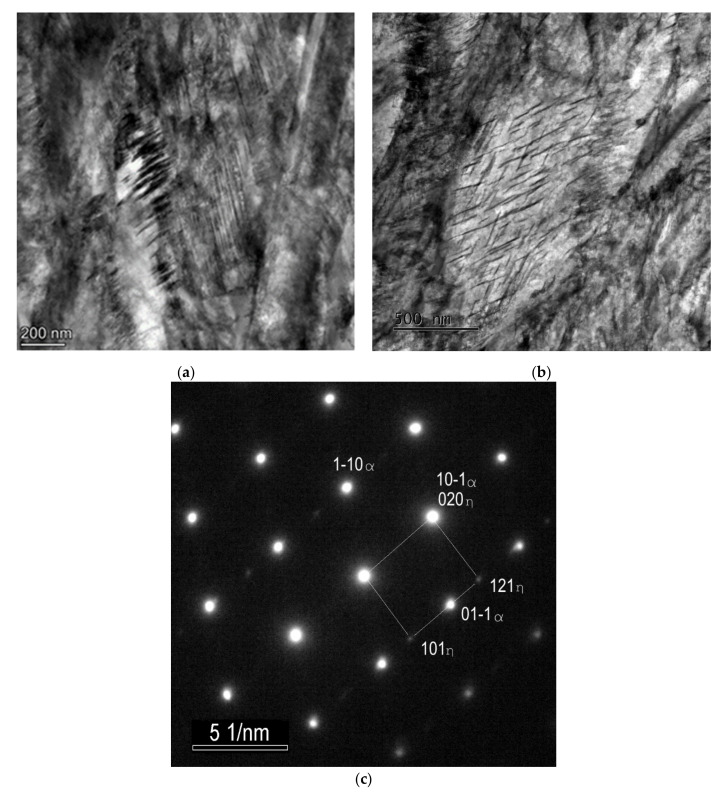
TEM micrographs of the sample after SAT350 processing, showing the interior of the bright island in martensite (**a**), the lath with η-Fe_2_C transition carbides (**b**), selected area electron diffraction (SAED) pattern of η-Fe_2_C carbides with zone axis [–101] (**c**).

**Figure 7:**
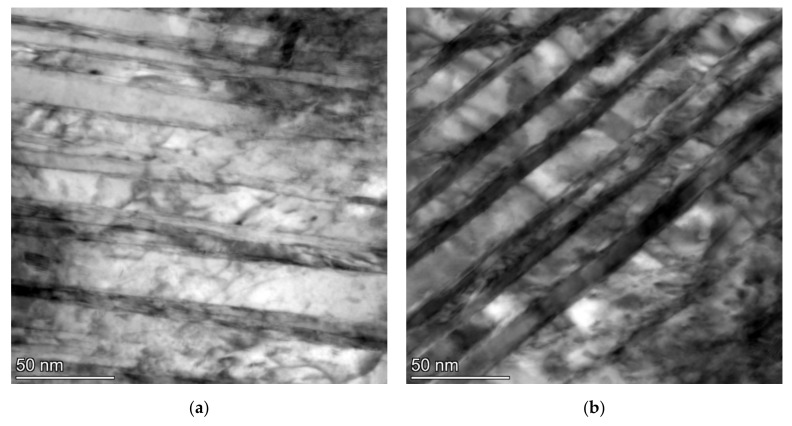
TEM micrographs of the sample after SAT350 processing, showing the interior of the bright island in martensite (**a**) and the lath with tempering carbides (**b**).

**Figure 8:**
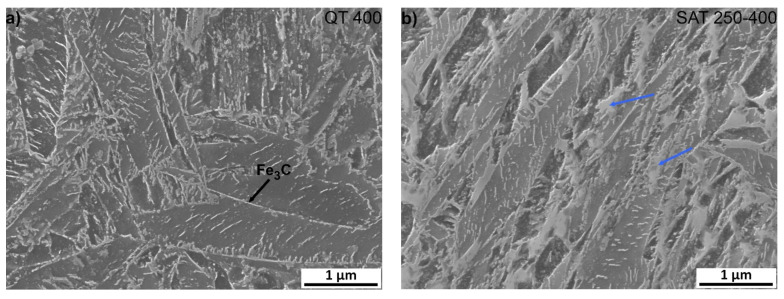
Microstructure analysis of samples after quenching and tempering at 400 °C, (**a**) QT and (**b**) SAT processing. Blue arrows indicate bright islands with the lower etchability.

Microstructural evolution during tempering and SAT treatment is shown in Figure 4, Figure 5, Figure 6, Figure 7 and Figure 8. Martensite laths up to 500 nm in width and length of several μm length occupy the whole volume of the sample just after quenching. The martensite laths’ geometric characteristics did not change due to QT or SAT. The size of PAG (12 μm) also did not vary. Twins of several nm thicknesses are visible at numerous localities after quenching. Almost no carbides are present in the microstructure, apart from exceptional small particles which arose because of non-zero diffusion during quenching.

Microstructures of samples tempered at 200–350 °C (QT 200–QT 350) and SAT treated (SAT 250–200 SAT 250–350) can be characterized by the tempered lath martensite with a high density of transition carbides, see Figure 5. The morphology of transition carbides seems to be very similar after both processing types at a tempering temperature of 200 °C. Nevertheless, it is possible to see the beginning of transition carbide dissolution into bright islands in SAT200 samples. The phenomenon of transition carbides decomposition into bright islands after SAT processing continues. It seems there is a slightly higher amount of that constituent in the microstructure as the tempering temperature of the second tempering increases. The area of bright islands reaches the highest area fraction in SAT 250–300 and SAT 250−350 samples. The blue arrows indicate examples of such localities. These islands have lower etchability compared to the surrounding matrix.

Additionally, numerous residual austenite islands are typically located at martensite lath boundaries, often at triple junctions of laths of both QT and SAT samples. The morphology of these islands is represented by relatively robust units (polygons), usually elongated in the direction of the adjacent martensitic laths. Sometimes it is challenging to distinguish residual austenite islands and the bright islands arising from the dissolution of the transition carbides. Therefore, the residual austenite formation tends to be more sharply demarcated and creates simple geometry units. The retained austenite content in the range from 3.5 to 4.5 vol.% was determined in samples with tempering temperatures up to 350 °C using XRD analysis.

Nanotwins filling the volume of the martensite laths were observed using TEM in the SAT 250−350 sample (Figure 6a). The frequency of twinned areas seems to be considerably higher than in the martensitic microstructure after quenching. No carbides are visible in the bright island formations.

A martensite lath with transition carbide formation is shown in Figure 6b. These transition carbides were identified as hexagonal η-Fe_2_C carbides using selected area electron diffraction pattern (SAED), see Figure 6c. The carbides create very orderly systems of thin sticks with a strong crystallographic relation to the matrix. The microstructure of the martensitic laths with tempering carbides after SAT processing seems to be very similar to the sample after QT processing.

The twinned area consists of thin twins with a relatively smooth appearance, a thickness of several nm and a slightly thicker intertwin space (around 25 nm) with some dislocation structures. However, dislocation density does not look very high in these intertwin spaces (Figure 7).

It is very significant for both samples that transition carbides show signs of decomposition; usually, the long lamellas of transition carbides decompose into shorter formations during tempering at 400 °C (Figure 8), and at the same time, cementite film precipitates along the grain boundaries of the martensite laths. Furthermore, after SAT processing, the formations of bright islands in the sample change their appearance slightly and start decomposition. Their total amount is probably slightly lower than in the sample after SAT 350. According to XRD results, the decomposition of retained austenite occurs above the tempering temperature of 350 °C and contents below 2 vol.% were evaluated in QT 400 and SAT 250–400 samples.

**Figure 9:**
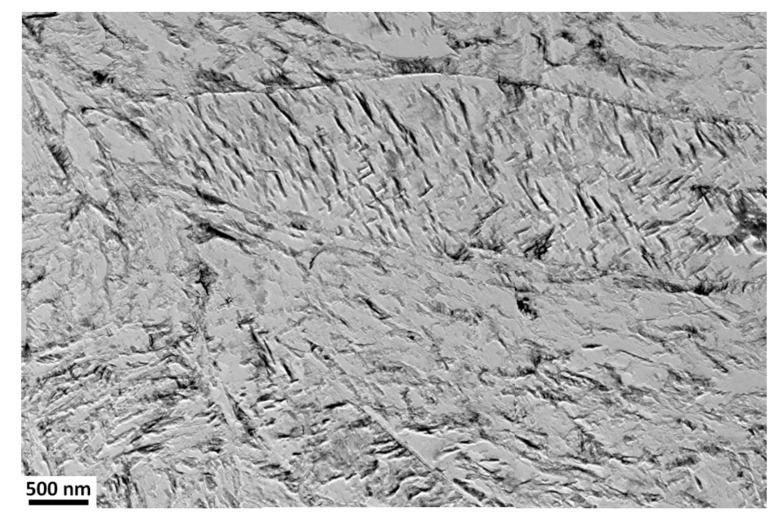
SAT 250–350, TEM carbon replica with extracted carbides.

TEM imaging and electron diffraction analysis on carbon extraction replicas were performed to specify different precipitates arising during tempering in the temperature range of 200–400 °C. The QT 250, QT 350, SAT 250 and SAT 350 samples were analyzed using this (Figure 9). Several phases (types of particles) were successfully extracted into the carbon replicas and then identified. The most common were Fe_2_C-ε (hexagonal), Fe_2_C-η (orthorhombic), Fe_5_C_2_-χ (monoclinic) and Fe_4_C (tetrahedral) carbides. 

**Figure 10:**
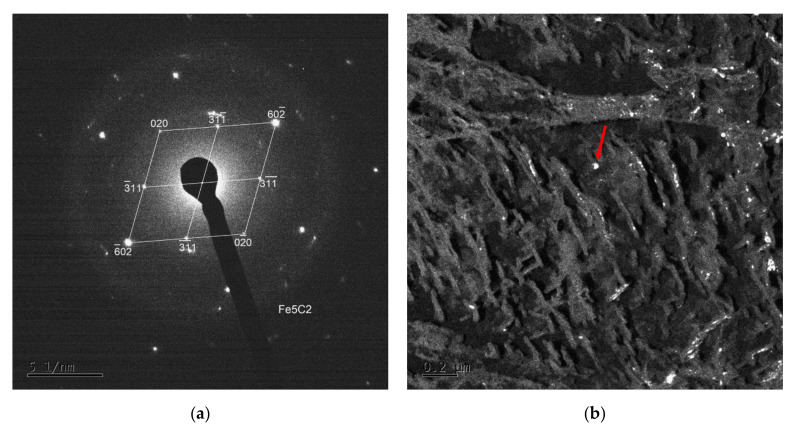
Selected area electron diffraction (SAED) pattern (**a**) and dark field mode of an extracted particle of Fe_5_C_2_ Hägg carbide (indicated by the red arrow) diffracting in the dark field (**b**).

Elongated lamellar-like carbides of Fe_2_C can be found in all samples where the carbon replica analysis was performed. Two variants—Fe_2_C-ε and Fe_2_C-η carbides—were found in QT250. In other samples, only one type—Fe_2_C-ε was found. Furthermore, many smaller, almost equiaxial particles were observed in all the samples. These particles were identified as Fe_5_C_2_ (Hägg carbide) or transition Fe_4_C (Figure 10).

**Figure 11:**
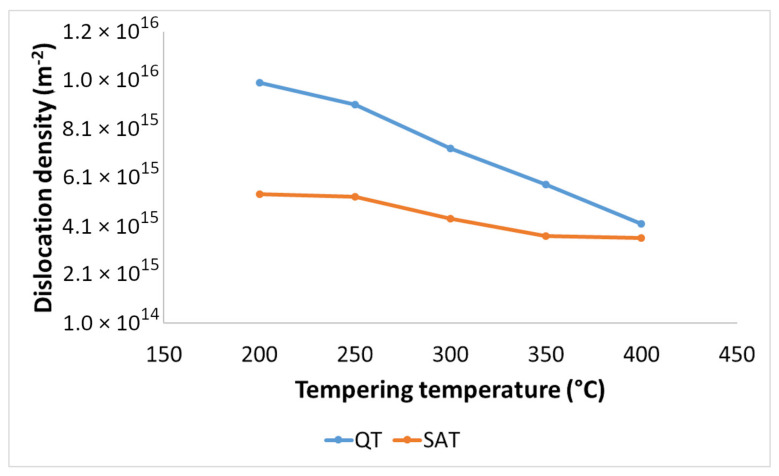
Dislocation density in QT and SAT samples tempered at different temperatures.

The dislocation density-tempering temperature diagrams for QT and SAT materials are depicted in Figure 11. Both curves show decreasing trends as the tempering temperature increases. Lower values of dislocation densities were determined for samples treated by the SAT regime. The difference between dislocation densities of QT and SAT samples decreased with increasing tempering temperature.

### 3.3. Dilatometry

The dilatometric experiments completed the whole experimental program by monitoring the tempering heat treatment processes. The experiments with continuous heating highlighted temperatures at which dominant diffusion processes are activated. The isothermal experiments helped to deepen the understanding of the entire course of the designed technological process. The samples (cylinders with diameters of 4 mm and lengths of 10 mm) were treated in the L78 RITA quenching dilatometer produced by Linseis.

There were three types of the initial state of dilatometric samples:

- Just after quenching;
- After quenching and first tempering (at 200 °C or 250 °C);
- After quenching, first tempering (at 200 °C or 250 °C) and controlled strain of 17%.

The initial state and labelling of all dilatometric samples are reviewed in Table 6.

### 3.4. Continuous Heating Tests

**Figure 12:**
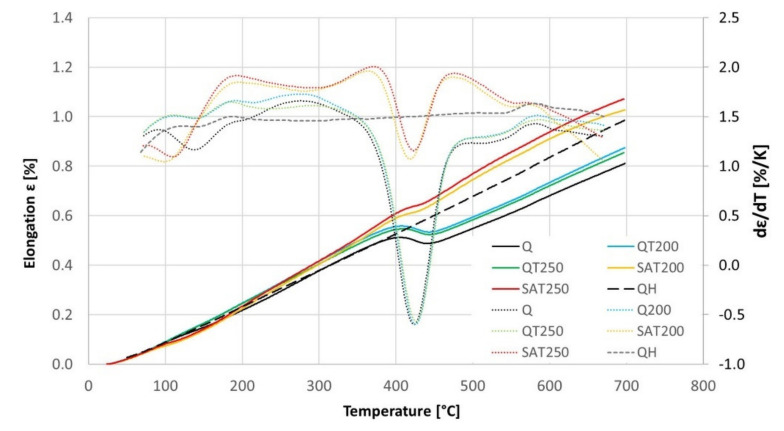
The elongation (complete lines) and its temperature derivative (dotted lines) with continuous heating of BX steel pretreated samples, according to Table 6. The dashed curves represent the second heating of quenched samples of BX steel.

The results of the continuous heating experiment are shown in the graph in Figure 12, in which the black curves represent the heating of the initially quenched sample: The precipitation of transition carbides in the temperature interval 100–180 °C and precipitation of cementite in the range 360–480 °C is visible as drops on the elongation curve and as peaks on the elongation derivative curve.

The blue and green curves show the heating of formerly tempered samples without embedded strain. In this case, the transition carbide formation was suppressed, while the cementite formation proceeded the same way as for just-quenched samples. This is because most of the transition carbides were formed during the first isothermal tempering during the preparation of the dilatometric samples. Therefore, the difference between the blue and green curves in the range of 200–300 °C is related to the different previous tempering temperatures.

The yellow and red curves record the heating of samples, which were tempered during preparation and deformed after quenching. These curves show that the transition carbide formation was strengthened and shifted to a lower interval from 80 to 150 °C. As a result, cementite precipitation was suppressed compared to the heating of the just-quenched samples. Furthermore, an important phenomenon is the strong increase of their derivative curves immediately after transition carbide precipitation in the range of 130–190 °C. This increase is considerably higher than the curves of samples without strain.

No difference was observed for samples deformed during preparation changing the tempering temperature before deformation. The course of the yellow curve is very similar to the red curve.

At temperatures above the range of transition carbide precipitation, all the dilatometric curves show increased lengths due to thermal elongation and residual austenite decomposition. This increase in length before cementite precipitation is remarkably higher for the deformed samples and is also shifted to higher temperatures.

Nevertheless, during continuous heating, all the dilatometric curves reveal a second length decrease or at least a slower length increase. After this slight reduction in length, there is an increase before a massive decline related to cementite precipitation.

**Figure 13:**
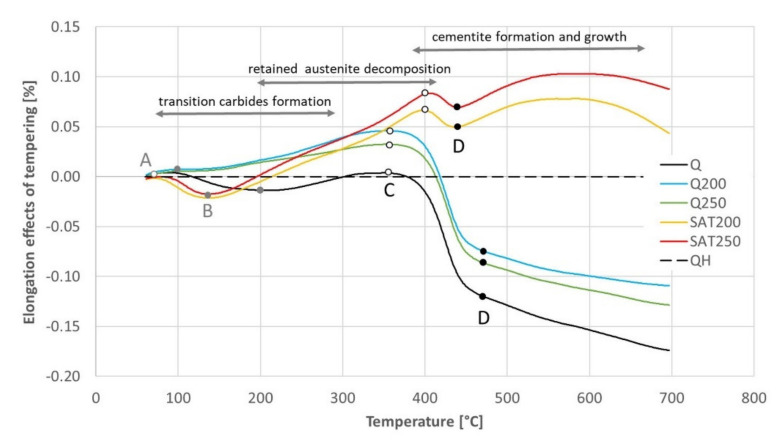
The difference in elongation between the sample without any transformation and other samples tempered with continuous heating. Points A, B, C and D were input data for Table 7.

The black dashed curve in Figure 12 shows the elongation of the sample without any transformation during tempering. The sample for this experiment was prepared by previous continuous heating up to 700 °C. This elongation curve is compared with all the others in Figure 13, and the dilatometric effects of the assumed individual processes are quantified in Table 7.

No signs of transition carbide formation were observed after isothermal tempering (blue and green curves). During continuous heating of the tempered and deformed sample (yellow and red curves), the length decreased in the corresponding region between points A and B in the same range as for the hardened sample (black curve) but at lower temperatures.

The dilatometric effect of cementite formation between points C and D was nearly ten times smaller for samples after SAT than for any other sample.

The elongation of the deformed samples increased markedly between points B and C (and even above the range of cementite formation). Such a significant increase has to be explained by a process other than only the austenite decomposition.

### 3.5. Isothermal Tests

**Figure 14:**
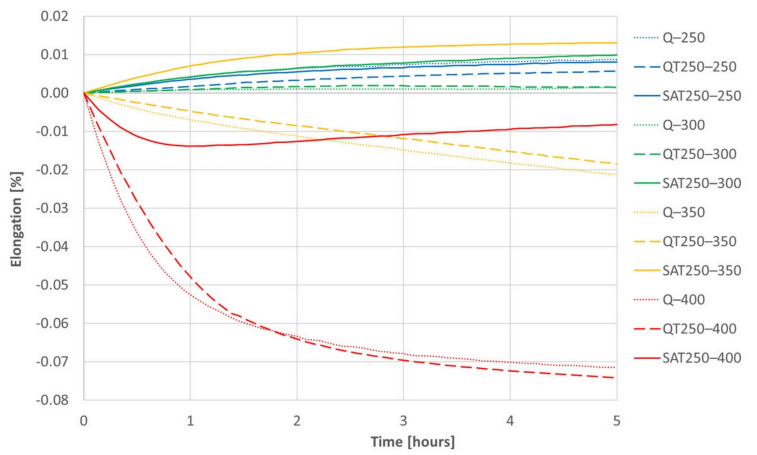
The series of dilatometric records of isothermal tempering of samples after quenching (dotted lines) or quenching and one tempering (dashed lines) or quenching, tempering and deformation (solid lines).

**Figure 15:**
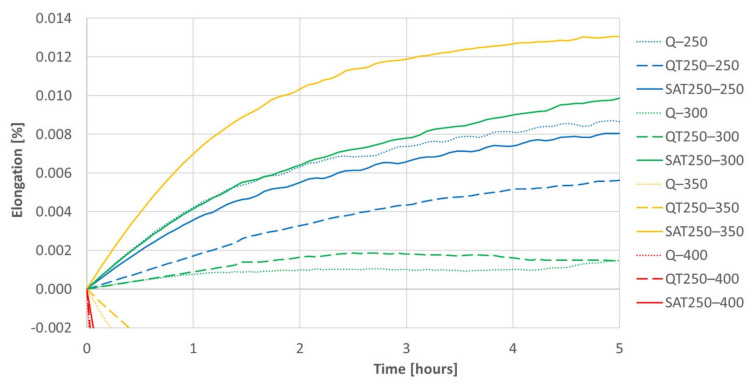
A detail of Figure 14 for positive elongation increments during isothermal tempering. The differences between samples processed with deformation and without are significant. These differences increasing with increasing temperature of the second tempering can be followed on the graph.

The results of isothermal dilatometric tests are shown in Figure 14 and Figure 15. Initial states for the isothermal tests were prepared the same way as for the continuous heating dilatometry tests.

The increasing trend of most curves is assumed to be due to retained austenite decomposition and general thermal elongation. This should overbalance the transition carbide precipitation, which causes the shortening of the sample, usually in the temperature range of 200–350 °C. However, the decrease in length due to carbides formation prevails only at 400 °C, when a massive formation of cementite arises. Therefore, a trend reversal from decreasing to increasing in the deformed sample tempered at 400 °C (red curve) corresponds to the suppression of cementite formation, which was also observed in the deformed samples during continuous heating.

Both figures represent the effect of the strain on the isothermal tempering during SAT processing. A comparison of all the records shows that the influence of the strain very distinctly increases with increasing second tempering temperature. The curves representing the behavior of SAT T1–T2 samples show a distinctly more significant increase in length than QT T1–T2 samples. The difference is striking, especially for the second tempering temperature of 350 °C. During the double tempering of QT 250–350, the intensive precipitation of transition carbides declines down the slope of the dilatometry record (yellow dashed line). On the other hand, the dilatometry record reveals the steepest climb (solid yellow line) for the second tempering of SAT 250–350. Such a difference in the dilatometry data can be hard to explain only in terms of changes in the austenite decomposition kinetics.

A comparison of the QT 250–400 and SAT 250–400 pair is interesting. The QT sample reveals massive cementite precipitation and a significant decrease in length (dashed red line in Figure 14). On the other hand, the dilatometry curve of SAT 250–400 drops at first as well; nevertheless, its course changes after 50 min of tempering and then the tendency is towards a slight increase. This means slower precipitation phenomena is overbalanced by other phenomena, which causes the length of the sample to increase. 

The dilatometry effects of the first and second tempering without included strain are very similar. 

## 4. Discussion

The materials’ phenomena in 54SiCr6 steel exposed to different variants of QT processing and SAT processing were compared in experimental programs using mechanical testing, metallography analysis, dilatometry measurement and X-ray analysis.

### 4.1. Development of Microstructure Features

A significant difference in mechanical testing values between QT samples and SAT samples indicates an essential distinction in the final microstructures and the physical processes leading to the final states of the samples. The processing history of QT and SAT samples takes different paths after the quenching and first tempering. In this state, the microstructure includes tempered martensite; therefore, there are martensite laths with transition carbides throughout the sample. Some laths contain areas with deformation twins arising during or just after quenching. During an applied 17% strain, the martensite laths undergo a fundamental change; many lattice defects probably occur in the SAT samples. The X-ray measurements did not reveal a significant enhancement of the dislocation density.

Nevertheless, other types of defects such as vacancies or planar defects may occur. There were also different microstructural processes in the second and first tempering. The precipitation of transition carbides has a similar intensity, but in SAT samples it is shifted to lower temperatures. This process even starts at a temperature as low as 100 °C, and its maximum intensity evaluated from the discontinuous dilatometry record was detected at 120 °C. With an increasing second tempering temperature, the precipitation process ceases to be dominant, and other processes lead to volume increase overbalance. One reason for volume increase could be the decomposition of residual austenite, but the volume increase during the second tempering of the deformed samples exceeds the expected value. A notable increase in volume during tempering after deformation is confirmed by both isothermal and anisothermal dilatometry. Besides residual austenite decomposition, other parallel phenomena probably occur during heating. Electron microscopy analysis revealed the transformation of tempered martensite matrix into bright islands in the SAT samples. This process, including transition carbide dissolution, could contribute to the detected volume increase. The structure of the bright islands is not described in detail. Nevertheless, according to the current electron microscopy results, the bright islands are carbideless. They contain many twinned areas with a nanotwin thickness of less than 10 nm and an intertwin space of less than 25 nm. The appearance of twins in SAT samples may be due to a high strain rate (120 s^−1^) in our study; according to [14,15,16,17,18], where it is stated that the tendency for mechanical twinning in BCC and HCP metals is quite strong at high strain rates and low temperatures. Subsequent second tempering results in the evolution of the twin population. This effect is close to its maximum after a second tempering at 350 °C. The dislocation density in twinned areas is not very high. The highest number of bright islands was detected by microscope after second tempering at 350 °C/2 h, where this modification of the martensitic phase occupies approximately 30% of the micrograph area. This finding corresponds to the isothermal dilatometry measurement, where tempering after deformation at 350 °C shows the most significant increase in the sample volume. The volume difference between the sample tempered at 350 °C after deformation and the sample tempered at the same temperature without deformation represents 0.03% of sample length. It illustrates very considerable value.

The presence of bright islands could also be related to the lower cementite precipitation after SAT processing. The dissolution of transition carbide into the martensitic matrix with simultaneous formation of cementite during tempering is well-known [19]. The precipitation of transition carbide and cementite is considered to be independent [20]; however, since the volume concentration of carbon in the matrix in equilibrium with the metastable transition carbide is higher than that of cementite, the transition carbides are dissolved [20], according to the classic nucleation theory [21]. In our case of pre-strain with a high strain rate, the twin boundaries appear, absorbing a significant amount of carbon. Thus, carbon depletion in the matrix occurs, and transition carbides dissolve. The carbon absorbed in the twins has activity that is too low to participate in the cementite precipitation reaction.

The increasing course of the samples processed with 17% strain above point D visible in the red and yellow curves in Figure 13 is quite interesting. An explanation of this increase is outside the scope of this work; nevertheless, it is essential to note it.

The bright islands with carbideless areas were not detected after standard QT processing in experimental steel.

### 4.2. Physical Mechanism for Changing Mechanical Properties

The strength parameters increased very significantly immediately after the applied strain. This is because the material just after deformation is very strong and has very low plasticity. Some deformation strengthening is responsible for this enhancement of the strength parameters. The intrinsic strength of the matrix, solid solution strengthening, precipitation strengthening, dislocation strengthening and grain boundary strengthening are analyzed for the lath martensite microstructure in [1,22]. The most relevant factor is the strengthening of grain boundaries since the chemical composition is the same, and the density of dislocations and precipitates’ parameters in the QT and SAT samples are close. The lath’s martensite, PAG boundaries, packet boundaries, block boundaries, and twins could provide the grain boundaries effect. In [2], it was proved that lath’s martensite plays a dominant role in MCLA steels in the absence of twins. Note that the twins’ effective grain size (EGS) was about 25 nm in SAT 250–350, while the EGS of martensite lath martensite was about 500 nm in both QT and SAT samples. Hence, it can be concluded that the formation of nanotwins by SAT is responsible for the increase in yield strength.

The tempering after deformation causes a slight improvement of elongation and reduction of the area. Still, yield and tensile strength remain very close if second tempering is carried out at low temperatures as was found for samples SAT 200–200, SAT 250–200, SAT 250–250 and SAT 250–300 (Table 5). A similar effect was observed in [23]. A second tempering increased the difference between yield and tensile strength at a second tempering temperature above 300 °C. A substantial improvement in the notch toughness parameter was observed with SAT. Notch toughness values are higher after SAT processing than QT processing, sometimes even more than 30% higher. Furthermore, the energy integrated below the curve of the force-deflection diagram (Figure 4b) is significantly higher. The material after SAT processing seems less sensitive to tempering embrittlement in the temperature range of 250–400 °C compared to QT processed samples (Figure 4a). It is possible that carbideless martensite in the bright islands could be responsible for such an improvement in the notch toughness. 

## 5. Conclusions

A comparison of QT and SAT processing of 54SiCr6 spring steel shows significant differences in mechanical properties, microstructures and dilatometry behavior, as well as in X-ray diffraction results. In addition, all tempering processes performed during QT and SAT processing were performed in the temperature range of 200–400 °C.

The basic influence on the differences between both types of processing is the 17% applied strain with a high strain rate, which causes immense changes in mechanical properties and the tempered martensite microstructure. Substantially different phenomena occur due to the first tempering of the as-quenched material and subsequent deformation compared to the conventional QT treatment. Precipitation of carbides is shifted to lower temperatures, and the speed of decomposition of austenite increases and is also moved to lower temperatures. The significant expansion of the volume of the heated samples after deformation implies the presence of phenomena which are not typical for tempering processes. These phenomena include the dissolution of transition carbides and the formation of carbideless martensite with nanotwin areas. The possible reason for the occurrence of twins in SAT samples is a high strain rate (120 s^−1^) of pre-strain by swaging. At the same time, the absence of carbides results from carbon depletion in the matrix caused by the carbon diffusion into the twin boundaries.

After SAT processing of 54SiCr6 steel, the mechanical properties show a very high increase for both tensile and yield strength. Many samples measured within the experimental program achieved values for YS and TS in the range 2500–3000 MPa. The formation of nanotwins due to SAT is responsible for increasing the strength parameters. Although plastic parameters decreased, the possibility also exists here to achieve interesting values. Promising results were observed during the measurement of notch toughness. All the notch toughness results measured after SAT processing were higher than the values measured after QT processing. The notch toughness does not decrease during tempering in the temperature range of 250–400 °C after SAT processing, but after QT processing, it does.

To optimize the strength and plastic properties of 54SiCr6 spring steel, we can recommend conventional quenching and tempering at a temperature of 250 °C for 2 h, supplemented by pre-strain deformation of 17% by swaging with a strain rate of 120 s^−1^ and a second tempering at 300 °C for 2 h.

## Figures and Tables

**Table 1:** The steels’ chemical compositions and standard 54SiCr6 according to ČSN EN 10 027-1, wt%.

Steel (wt%)	C	Si	Mn	Cr	Cu	Fe
BX	0.57	1.5	0.68	0.75	0.12	Bal.
54SiCr6	0.51–0.59	1.2–1.6	0.5–0.8	0.5–0.8	-	Bal.

**Table 2:** Quenching and tempering processing.

Quenching Temperature (°C)/ Holding Time (h)	Tempering Temperature (°C)/ Time of Tempering (h)	Designation of the Sample
900/20	-----	QT000
900/20	200/2	QT200
900/20	250/2	QT250
900/20	300/2	QT300
900/20	350/2	QT350
900/20	400/2	QT400

**Table 3:** SAT processing.

Quenching Temperature (°C)/ Holding Time (min)	1st Tempering (°C)/(h)	Strain (%)	2nd Tempering (°C)/(h)	Designation
900/20	200/2	17	---	SAT 200
900/20	200/2	17	200/2	SAT 200−200
900/20	250/2	17	200/2	SAT 250−200
900/20	250/2	17	250/2	SAT 250−250
900/20	250/2	17	300/2	SAT 250−300
900/20	250/2	17	350/2	SAT 250−350
900/20	250/2	17	400/2	SAT 250−400
900/20	250/2	17	300/6	SAT 250−300/6
900/20	250/2	17	300/10	SAT 250−300/10

**Table 4:** Results of tensile testing, conventional quenching and tempering (QT).

Sample Designation	YS (MPa)	UTS (MPa)	EL (%)	AR (%)	KCV (J/cm^2^)
QT200	1654 ± 4	2439 ± 1	4.7 ± 0.7	4.3 ± 2.7	19.7 ± 1.5
QT250	1893 ± 8	2277 ± 20	6.3 ± 1.4	25.2 ± 3.5	18.0 ± 1.6
QT300	1915 ± 7	2241 ± 9	8.3 ± 0.4	34.1 ± 3.0	15.3 ± 1.8
QT350	1912 ± 1	2167 ± 1	9.2 ± 0.1	39.3 ± 2.8	12.1 ± 0.1
QT400	1705 ± 5	1897 ± 4	10.3 ± 0.5	43.9 ± 0.1	21.4 ± 1.8

**Table 5:** Results of tensile testing, quenching and strain-assisted tempering (SAT).

Sample Designation	YS (MPa)	UTS (MPa)	EL (%)	AR (%)	KCV (J/cm^2^)
SAT 200−200	2984 ± 4	2990 ± 3	-	7.7 ± 0.4	14.8 ± 3.5
SAT 250−200	2836 ± 38	2837 ± 37	2.0 ± 0.4	15.9 ± 3.9	20.8 ± 2.4
SAT 250−250	2753 ± 21	2759 ± 25	3.7 ± 0.8	16.3 ± 2.7	20.7 ± 1.0
SAT 250−300	2709 ± 16	2717 ± 18	4.1 ± 0.6	20.4 ± 2.4	19.2 ± 0.5
SAT 250−350	2491 ± 13	2511 ± 7	4.1 ± 0.1	19.3 ± 2.0	15.7 ± 0.4
SAT 250−400	2036 ± 9	2091 ± 7	8.0 ± 0.1	36.7 ± 1.5	22.4 ± 0.2
SAT 250−300/6	2601 ± 14	2612 ± 13	5.4 ± 0.9	20.8 ± 5.3	19.2 ± 1.5
SAT 250−300/10	2619 ± 26	2630 ± 22	4.9 ± 1.0	30.8 ± 1.4	17.4 ± 1.7

**Table 6:** The initial state of dilatometric samples studied with continuous heating and isothermal tempering.

Initial State	Continuous Heating Tests	Isothermal Tests				
		200 °C	250 °C	300 °C	350 °C	400 °C
	Sample Designation					
as quenched	Q	Q200	Q250	Q300	Q350	Q400
Tempered (200 °C/2 h)	QT 200	QT 200–200	QT 200–250	QT 200–300	QT 200–350	QT 200–400
Tempered (250 °C/2 h)	QT 250	QT 250–200	QT 250–250	QT 250–300	QT 250–350	QT 250–400
tempered (200 °C/2 h) +17% strain	SAT 200	SAT 200–200	SAT 200–250	SAT 200–300	SAT 200–350	SAT 200–400
tempered (250 °C/2 h) +17% strain	SAT 250	SAT 250–200	SAT 250–250	SAT 250–300	SAT 250–350	SAT 250–400

**Table 7:** The differences in elongation read from Figure 13 at points before the main effects of transition carbides formation (column A), retained austenite decomposition (B), cementite precipitation (C) and just above the main effect of cementite precipitation (D). The differences associated with the transformations are calculated in the columns between the readings.

Preparation		Assumed Dominant Dilatometric Effect							
		Transition Carbides			Austenite		Cementite		
		A		B			C		D
**Q**	quenching	80 °C 0.004%	−0.018%	200 °C −0.014%		+0.018%	360 °C 0.004%	−0.124%	470 °C −0.120%
QT200	temp. 200 °C	–	–	100 °C 0.005%		+0.043%	360 °C 0.048%	−0.122%	470 °C −0.074%
QT250	temp. 250 °C	–	–	100 °C 0.005%		+0.027%	360 °C 0.032%	−0.118%	470 °C −0.086%
SAT200	SAT 200 °C	80 °C 0.000%	−0.021%	140 °C −0.021%		+0.087%	400 °C 0.066%	−0.016%	440 °C 0.050%
SAT250	SAT 250 °C	80 °C 0.000%	−0.017%	140 °C −0.017%		+0.100%	400 °C 0.083%	−0.014%	440 °C 0.069%

## Data Availability

The data presented in this study are available on request from the corresponding author.
